# Comparison of serum zinc levels between patients with sinonasal neutrophilic and eosinophilic inflammatory polyposis and healthy individuals

**DOI:** 10.1002/iid3.70016

**Published:** 2024-11-26

**Authors:** Matin Ghazizadeh, Elahe Roshanaie, Behrouz Barati

**Affiliations:** ^1^ Department of Otorhinolaryngology, Head and Neck Surgery Taleghani Hospital, Shahid Beheshti University of Medical Sciences Tehran Iran

**Keywords:** eosinophil, inflammation, nasal polyps, neutrophil, polyps, zinc

## Abstract

**Objectives:**

Some changes in nasal mucus and paranasal sinuses may occur due to zinc deficiency, which can cause chronic rhinosinusitis with nasal polyps (CRSwNP). The current study was designed to compare the serum zinc concentration between patients with chronic rhinosinusitis complicated with eosinophilic or neutrophilic nasal polyps and a control group.

**Methods:**

A total of 105 patients participated in the study. Patients in three different groups were evaluated for CRSwNP (35 in the eosinophilia group and 35 in the neutrophil group), and 35 patients underwent surgery for reasons other than polyposis (control group). The serum zinc level was determined.

**Results:**

The mean age of the patients was 39.4 ± 12.61 years. Forty‐one patients (39%) were female. Based on the enzyme linked immunosorbent assay results, the average serum zinc level in the control group was 137.01 ± 19.42 (μgm/100 mL), and in all patients with CRSwNP, it was 127.27 ± 21.7 (μgm/100 mL). The serum zinc concentration in patients with CRSwNP was significantly lower than that in the control group (*p* = .027). Among the CRSwNP patients with eosinophilic polyps and neutrophilic polyps, 130.42 ± 21.92 (μgm/100 mL) and 127.27 ± 21.7 (μgm/100 mL), respectively, were detected. Based on the statistical analysis, the neutrophilic and eosinophilic groups were homogenous according to the average serum zinc concentration (*p* = .631), and the same conditions prevailed for the eosinophilic and control groups (*p* = .574). There was a noticeable distinction between the neutrophilic group and the control group (*p* = .034).

**Conclusion:**

Serum zinc concentrations were significantly lower in patients with neutrophilic polyps than in the general population. This difference may be due to the essential role of zinc in the inflammatory process in patients with neutrophilic polyposis.

## Key points

- The serum zinc level with Sinonasal Polyposis was lower than the control group.
- The neutrophilic and eosinophilic groups were homogenous in the mean zinc level.
- Zinc serum level is different between the eosinophil and control group.

## 1 INTRODUCTION

The specific underlying mechanism involved in the pathogenesis of chronic rhinosinusitis with nasal polyps (CRSwNP) is unknown. However, mucosal inflammation of the sinonasal cavities has been proposed to play a crucial role in its pathogenesis.^1^, ^2^ CRSwNP can occur at any age but is more common in young and middle‐aged people.^3^, ^4^ Inflammation is the primary factor that increases the possibility of CRSwNP.^5^ Other factors that can also affect the pathogenesis of CRSwNP are asthma, sensitivity to aspirin, cystic fibrosis, and family history.^6^ CRSwNP has different endotypes and phenotypes, and even when patients have similar phenotypes, histopathological examinations may reveal different endotypes, which may lead to differences in treatment strategies. In 90% of nasal CRSwNP patients, eosinophils and neutrophils are the most common cells. The concentration of these cells in the mucosa is directly related to the severity of the disease and the success of the treatment.^7^

Microminerals are a collection of metallic and nonmetallic elements. For the human body to operate correctly, small quantities of these substances are needed. Zinc is an essential nutrient that our bodies cannot produce or store. For this reason, we must provide it constantly through our diet. Zinc is involved in many processes in the body, including genetic processes, enzyme reactions, immune function, protein and DNA synthesis, and wound healing.^8^, ^9^ Zinc deficiency can cause changes in nasal mucus and the paranasal sinus and cause CRSwNP by inducing the mutual immune reaction of Th2 cells with eosinophils.^10^, ^11^, ^12^ Several studies have investigated the tissue level of zinc in patients with nasal polyps, but very few have explored the serum levels of this element in such patients. Several studies have investigated the tissue level of zinc in patients with nasal polyps, but very few have explored the serum levels of this element in such patients. Zinc deficiency is considered a possible etiology. This study aimed to compare the levels of zinc in the serum of patients with eosinophilic and neutrophilic polyps and those in the general population.

## 2 METHODS

The present case‐control study was conducted between February 2020 and February 2022 to compare zinc serum levels in patients with CRSwNP with those in the general population. Our study was approved by the Department of ENT and passed the procedures for obtaining the code of ethics in biomedical studies (IR.SBMU.MSP.REC.1399.686). All the steps, objectives, and nature of CRSwNP were explained to the patients. The patients signed the informed consent form and were subsequently included in the study. Overall, 105 patients were divided into three groups: 70 patients with CRSwNP (35 in the eosinophilic polyp group and 35 in the neutrophilic polyp group) and 35 healthy patients who underwent surgery for reasons other than polyposis (septorhinoplasty, septoplasty, blepharoplasty, tympanoplasty, or stapedectomy). So, we checked all of them preoperatively for possible sinonasal diseases. We collected the patients’ demographic information and medical history.

All patients with CRSwNP underwent diagnostic nasal endoscopy and paranasal sinus CT scans.

The inclusion criteria were age older than 18 years, functional endoscopic sinus surgery (FESS) candidate, no underlying disease, and no history of zinc supplement consumption in the last 3 months.

Patients were excluded from the study if the pathological examination was negative for something other than CRSwNP (like a tumor). The control group included healthy patients who underwent surgeries other than FESS.

In the operating room, 5 cc of blood samples were collected in special metal‐free tubes. The serum zinc concentrations were determined using atomic absorption spectrometry and total fluorescence simultaneously. At the end of the operation, the polyps were subjected to histological examination. We determined whether the tissue samples had a predominance of eosinophilic or neutrophilic cells. We subsequently analyzed the results of three groups, which included a control group, a group of patients with eosinophilic sinonasal polyposis (the eosinophilic group), and a group of patients with neutrophilic sinonasal polyposis (the neutrophilic group).

The following findings were collected: demographic characteristics of the individuals; medical history of diabetes, hypertension, smoking, or dyslipidemia; and biochemical measurements of the serum and pathological samples. The statistical analysis was performed using SPSS version 22 statistical software (SPSS, Inc.). The data distribution was evaluated using the Kolmogorov‒Smirnov test. The Chi‐square test and *t*‐test were used to compare qualitative and quantitative data. Additionally, analysis of variance (ANOVA) and Fisher's post hoc tests were applied to compare the three groups. A *p* value less than .05 was considered to indicate statistical significance.

## 3 RESULTS

The average ages of the patients in the case and the control groups were 39.4 ± 12.61 years and 39.09 ± 11.68 years, respectively. Based on the statistical analysis, there was no statistically significant difference in age (*p* = .449).

The demographic characteristics and medical history of the patients in terms of diabetes, hypertension, smoking status, and dyslipidemia status are shown in Table 1. According to the Chi‐square test, there were no significant differences among the three groups in terms of diabetes (*p* = .322), hypertension (*p* = .154), dyslipidemia (*p* = .792), smoking (*p* = .738), or sex (*p* = .852). Since the difference in the number of diabetic patients in the neutrophilic group was more significant than that in the other two groups, the confounding effect of having diabetes was adjusted for using a multiple linear regression model. We detected a significant difference between the neutrophilic group and the other groups (*p* = .005).

**Table 1:** Demographic and medical history of the study patients.

	Control group (*n* = 35)	Eosinophilic group (*n* = 35)	Neutrophilic group (*n* = 35)	Total (*n* = 105)	*p* value^a^
Age, mean (*SD*)	39 (11)	41 (13)	37 (12)	39 (12)	.449^b^
Female, *n* (%)	13 (37)	13 (37)	15 (42)	41 (39)	.852
Diabetes, *n* (%)	7 (20)	6 (17)	11 (31)	24 (22)	.322
Hypertension, *n* (%)	13 (37)	16 (46)	21 (60)	50 (48)	.154
Dyslipidemia, *n* (%)	6 (17)	4 (11)	5 (14)	15 (14)	.792
Smoking, *n* (%)	10 (29)	13 (37)	11 (31)	34 (32)	.738

^a^ Chi‐square test.

^b^ One‐way analysis of variance (ANOVA).

The average serum zinc levels in the control, eosinophilic, and neutrophilic groups were 137.01 ± 19.42 (μgm/100 mL), 130.42 ± 21.92 (μgm/100 mL), and 124.11 ± 21.40 (μgm/100 mL), respectively (*p* = .040) (Table 2). According to the ANOVA, there was a difference in the mean serum zinc concentration between at least one group and the others. In this field, we utilized the Bonferroni post hoc correction to eliminate any uncertainties, as reported in Table 3. There was no significant difference in the average serum zinc levels between the neutrophilic and eosinophilic groups. The same conditions prevailed for the eosinophilic and control groups, but an obvious difference was observed between the neutrophilic and control groups (*p* = .034).

**Table 2:** Zinc serum levels in the studied groups.

	Control group (*n* = 35)	Eosinophilic group (*n* = 35)	Neutrophilic group (*n* = 35)	*p* value^a^
Serum level of zinc (μgm/100 mL); mean ± *SD*	137.01 ± 19.42	130.42 ± 21.92	124.11 ± 21.40	.040

^a^ one‐way analysis of variance (ANOVA).

**Table 3:** Bonferroni post hoc test results to compare zinc serum levels in the three groups under study.

	Difference in the average level of zinc serum (confidence interval)	*p* value
Control group and Eosophilic group	6.5 (16.5, 3.3)	.574
Control group and Neutrophilic group	12.8 (22.8, 2.9)	.034
Eosinophilic group and Neutrophilic group	6.3 (16.2, 3.6)	.631

According to the results of the two‐sample independent *t*‐test, the serum zinc concentration of subjects with CRSwNP was significantly lower than that of the control group (*p* = .027) (Table 4).

**Table 4:** Serum levels of zinc in control and sinonasal polyposis patients.

	Control group (*n* = 35)	Sinonasal polyposis group (*n* = 70)	*p* value^a^
Serum level of zinc (μgm/100 mL); mean ± *SD*	137.01 ± 19.42	127.27 ± 21.74	.027

^a^ Two independent samples *t*‐test.

## 4 DISCUSSION

In the present study, we examined the possible relationship between the serum zinc concentration and CRSwNP incidence. It was lower in patients with CRSwNP than in the control group. This issue can be evaluated in different ways. The first issue can be due to the strong anti‐inflammatory role of zinc. Zinc is a potent mediator of cell‐dependent immunity and has anti‐inflammatory and antioxidant effects.^13^, ^14^ Inflammation plays a significant role in the development of polyps.^4^, ^5^ In our study, the amount of serum zinc was lower in the CRSwNP group than in the control group, which may indicate an increase in inflammation in the CRSwNP group. Nevertheless, to make a definite opinion about this phenomenon, it is necessary to measure the serum and tissue levels of inflammation‐related biomarkers, such as interleukins and tumor necrosis factor‐alpha and beta rays.

Another considerable issue is the antioxidant property of zinc and the crucial role of oxidative stress in the pathogenesis of CRSwNP.^15^, ^16^ It has been suggested that zinc can prevent cellular damage and destruction of cell membranes by converting hydrogen peroxide into less toxic products.^17^ Although we have enough information about the antioxidant effects of zinc, it is necessary to investigate various biomarkers related to antioxidant defense in CRSwNP patients, such as superoxide dismutase, catalase, glutathione peroxidase, and glutathione reductase; as in the present study, it was confirmed that the levels of zinc in this particular population were lower. This critical issue should be taken into consideration in future studies.

In the neutrophilic polyposis group, the amount of serum zinc was significantly lower than that in the control group; the possible explanation is that neutrophilic polyps are common under inflammatory conditions, while eosinophilic polyps are prevalent under allergic conditions. Zinc deficiency causes changes in nasal mucus and paranasal sinuses and causes CRSwNP through the induction of an immune response related to Th2 cells.^12^, ^18^ However, further in‐depth studies are necessary to form a definitive opinion on this matter. The results of our study emphasize the anti‐inflammatory role of zinc.

Very few studies have investigated the serum levels of zinc in patients with CRSwNP, and most have investigated the levels of zinc in the mucus and lamina propria. Suzuki et al.^19^ conducted a study to explore the use of metallothionein‐3 levels as a biomarker for assessing zinc levels in the nasal mucosa of patients suffering from rhinosinusitis with CRSwNP. The results of their study showed that in both the mucus and lamina propria of patients with CRSwNP, the expression level of metallothionein‐3 decreases, and there is a relationship between the level of zinc in the mucosal layer and the expression of metallothionein‐3. The findings of the present study were consistent with the results of their research. In 2021, a recent study led by Nikakhlagh and his team compared the levels of zinc and selenium in the tissues of patients with CRSwNP to those of healthy individuals.^20^ Their study showed that the tissue level of zinc in the CRSwNP group was significantly lower than that in the control group, but there was no significant difference in the tissue level of selenium. This finding was also consistent with the present study. However, our study had a much larger sample size than the previously mentioned study, which increased the strength of our findings (105 vs. 72).

A distinct characteristic of the current study was that the underlying diseases that could affect zinc metabolism or inflammatory pathways were considered in both case and control groups. Although there were no statistically significant differences in the prevalence of these diseases among the three studied groups, diabetes mellitus was more common in patients with neutrophilic polyps. Searching the web, we didn't find any proven correlation between CRSwNP and diabetes. It seems to be a novel field of research. However, the possible explanation is that due to high blood sugar, the immune system in diabetic patients does not work normally. Also, adipose cells and macrophages in the fat tissues, produce a large amount of inflammatory factors. These inflammatory mediators may play an important role in the formation of inflammatory neutrophilic polyps.^21^

Serum zinc levels are lower in patients with neutrophilic polyps than in the general population. Zinc gets involved in many processes, including genetic processes, enzyme reactions, immune function, protein and DNA synthesis, and wound healing.^8^, ^9^ Immunohistochemistry examination for important factors from both inflammatory mechanisms and DNA repair mechanisms seems to be an essential way to understand the significance of lowered serum zinc levels in the formation of inflammatory polyps. On the other hand, the probable mechanism of polyp formation in CRSwNP is type 2 inflammation. High levels of interleukin‐4, interleukin‐5, and interleukin‐13 are known immunologic features of this type of human body reaction. Recently, biological treatment has been recommended for severe CRSwNP, targeting these cytokines.^22^ Determining the level of zinc and other known inflammatory factors in the patients’ serum simultaneously is a logical way to reveal the actual role of zinc and its possible mechanisms in CRSwNP.

The main limitation of this study is the absence of data on zinc concentrations in nasal secretions and tissue zinc levels in nasal inflammatory and eosinophilic polyps, alongside serum zinc levels. Previous studies that reported lower zinc levels in the nasal mucosa and tissues of patients with CRSwNP did not concurrently measure serum zinc levels.^19^, ^20^ The specific mechanisms by which serum zinc levels contribute to the formation of inflammatory polyps remain unclear. Zinc deficiency may either play a primary role in polyp formation or occur secondarily due to zinc consumption in immune and inflammatory processes. A more comprehensive study that simultaneously examines zinc levels in polyp tissues, nasal secretions, and patient serum could address this scientific gap. Additionally, a double‐blind, randomized clinical trial to assess the impact of zinc supplementation on the treatment and prevention of recurrence in CRSwNP patients may prove beneficial.

## AUTHOR CONTRIBUTIONS

**Matin Ghazizadeh**: Conceptualization; investigation; project administration; supervision; visualization; writing—original draft; writing—review and editing. **Elahe Roshanaie**: Data curation; formal analysis; resources; software; validation; writing—original draft. **Behrouz Barati**: Data curation; software; supervision; visualization.

## CONFLICT OF INTEREST STATEMENT

The authors declare no conflicts of interest.

## ETHICS STATEMENT

This research was approved by the Institutional Ethics Committee at Shahid Beheshti University of Medical Sciences under the reference number IR.SBMU.MSP.REC.1399.686. The research was conducted ethically, with all study procedures being performed in accordance with the requirements of the World Medical Association's Declaration of Helsinki. Written informed consent was obtained from each patient for study participation and data publication.

## Data Availability

Not available.
